# Group 2 Innate Lymphoid Cells (ILC2): Type 2 Immunity and Helminth Immunity

**DOI:** 10.3390/ijms20092276

**Published:** 2019-05-08

**Authors:** De’Broski R. Herbert, Bonnie Douglas, Kelly Zullo

**Affiliations:** School of Veterinary Medicine, University of Pennsylvania, Philadelphia, PA 19104, USA; bonniebd@pennmedicine.upenn.edu (B.D.); kzullo@pennmedicine.upenn.edu (K.Z.)

**Keywords:** helminth, mucosa, type 2 immunity, innate lymphocyte

## Abstract

Group 2 innate lymphoid cells (ILC2) have emerged as a major component of type 2 inflammation in mice and humans. ILC2 secrete large amounts of interleukins 5 and 13, which are largely responsible for host protective immunity against helminth parasites because these cytokines induce profound changes in host physiology that include: goblet cell metaplasia, mucus accumulation, smooth muscle hypercontractility, eosinophil and mast cell recruitment, and alternative macrophage activation (M2). This review covers the initial recognition of ILC2 as a distinct cell lineage, the key studies that established their biological importance, particularly in helminth infection, and the new directions that are likely to be the focus of emerging work that further explores this unique cell population in the context of health and disease.

## 1. Introduction

Type 2 inflammatory responses are elicited in hosts infected with parasitic helminths or exposed to xenotoxic agents, venom, and allergens [1,2]. While previously considered that CD4^+^ T cells and certain myeloid populations were the predominant sources of Type 2 cytokines, the past decade has been met with strong evidence that a rare subset of innate lymphocytes are major sources of type 2 cytokines, including interleukin 5 and 13. Originally described as non-B, T, myeloid, or natural killer (NK) cells elicited by helminthic parasites, it is now well accepted that innate lymphoid type 2 cells (ILC2) are a major component of type 2 immunity in mice and humans that are crucial for immunity against helminths.

## 2. Historical Perspective

These cells were first identified in 2006 as a non-B, non-T (NBNT) IL-13 producing cell that was dependent on the production of IL-25 [3]. Interestingly, NBNT cells were specifically found in the mesenteric lymph node (MLN), but not the spleen or bone marrow, and expanded in the presence of IL-25 as well as during day five of *Nippostrongylus brasiliensis* (*N. brasiliensis*) [3]. Furthermore, this subset of NBNT cells arose when recombinant IL-25 was administered intraperitoneally (i.p.) in RAG1^−/−^ and IL4^−/−^IL5^−/−^IL9^−/−^IL13^−/−^mice, suggesting that this population of cells arose independently of T and B cell lineages and type 2 cytokines [3]. In 2010, these cells were further described by three independent groups as lineage negative CD45^+^ ST2^+^ IL17BR^+^ that variably expressed Sca-1 and c-Kit and produced large amounts of IL-5 and IL-13 [4,5,6]. Each group proposed a different name for these cells. Moro et al. suggested “natural helper cells”, Neill et al. coined “nuocytes” for the 13th letter of the Greek alphabet, nu, and Price et al. recommended “innate type 2 helper cells” [4,5,6]. However, studies consistently demonstrated that IL-13 from these cells was necessary for expelling *N. brasiliensis*, because recipients of adoptively-transferred IL-13-deficient nuoctyes/natural helper/innate type 2 helper cells did not expel *N. brasiliensis* [7]. These cells were also found at the steady state in the MLN, spleen, lung, peritoneum, liver, and bone marrow in naïve mice and were lineage^negative^ c-kit^low^ Sca-1^−^ IL2rβ^low^ Ly5.2^+^ Thy1^+^ and CD44^high^ [5]. While this population was preserved in Rag2^−/−^ mice, it was absent from Rag, common γ chain double knock-out mice, suggesting that the γ chain, a key component of IL-2, 7, 9, and 15 receptors, was necessary for the development and survival of nuocytes [5,8]. All of these reports consistently identified an innate cell source of IL-13 that was expanded in the presence of IL-25, IL-33, or *N. brasiliensis* infection. Finally, in 2013, Spits et al. proposed that these cells be universally referred to as group 2 innate lymphoid cells (ILC2) [9]. A summary of mice and human ILC2 markers is listed in Table S1. 

## 3. ILC2 Development

ILC2s are found at mucosal tissue sites, including the lung, small intestine, colon, and MLN, as well as in the bone marrow, spleen, liver, kidney, and adipocyte tissue [10,11]. It is now widely accepted that ILC2s express CD90 and IL7R^alpha^ [4,8,12], and that their development and function rely on transcription factors GATA3, RORα, Id2, and NFIL3 [13]. ILCs develop from a common lymphoid progenitor, followed by a lymphoid progenitor that is α_4_β_7_^+^, a common helper-like ILC progenitor (CHILP), and finally differentiate into ILC2 progenitor cells (ILC2p) [14].

ILC2 progenitor cells (ILC2p) are found in the bone marrow and are Lin^−^Sca1^hi^GATA3^hi^ CD90^+^ IL7R^+^ID2^+^IL2rg^+^cells that produce low amounts of IL-5 [15]. ILC2p cells were identified as specific progenitors for ILC2 because, when cultured with OP9 feeder cells or Notch ligand delta like 1 (OP9-DL1) expressing OP9 cells in the presence of IL-7 or IL-7 plus IL-33, these cells expressed ILC2 transcription factors Id-2 and Gata3, but not markers for T or B cells [16,17]. To further understand what drives the progression of ILC2 development, GATA3 was deleted in all hematopoietic cells using Gata3^fl/fl^ vav-cre mice. This deletion led to the loss of ILC2p cells in the bone marrow and ILC2 cells from the small intestine and lung, suggesting that GATA3 is important for both the development and maintenance of ILC2 [18]. In addition to GATA3, there is a cell intrinsic role for RORα expression in the development of ILC2 cells. Whereas ILC2s can develop from RORα-sufficient bone marrow when co-cultured with OP9-DL1 cells, ILC2s do not arise from RORα^sg/sg^ bone marrow under the same conditions [19].

Maturation of ILC2p requires additional transcription factors. T-cell specific high mobility group box transcription factor (TCF-1) is a transcription factor that is required for ILC2 development [20]. *Tcf7* (encodes for TCF-1) mice had reduced numbers of ILC2s in the lungs and a statistically significant loss of ILC2p in the bone marrow compared to wild-type WT mice [20]. These data suggest that TCF-1 is required for ILC2p and ILC2 development. In addition, it has been shown that *Tcf7* promotes ILC2 development in a GATA3 dependent-manner through the upregulation of IL17rb and IL2Ra. Tcf1 can also promote ILC2 development in a GATA3 independent manner through the upregulation of IL7Ra expression [20].

In addition to the transcription factors that control ILC2 development, the cell adhesion molecule intercellular cell adhesion molecule (ICAM1) is enhanced on all ILC2p, immature, and mature ILC2 cells and promotes type 2 immune responses when bound to its ligand, LFA-1 [11]. Furthermore, ICAM1 has an important role in ILC2 development and function inasmuch as *Icam1* deficient (ICAM1^−/−^) mice have a decrease in both immature and mature ILC2 frequencies and number in the lung, intestine, and bone marrow [11]. Additionally, following a generation of mixed chimeras, the majority of ILC2s that developed were from ICAM1-sufficient CLP and not ICAM1^−/−^ CLP [11]. Collectively, these data suggest that ICAM1, TCF7, RORα, NFIL3, and GATA3 are all essential for the development of ILC2. However, ICAM and TCF7 deficiencies do not completely diminish all production of ILC2 development, therefore understanding how a subset of ILC2s develop in the absence of these genes is an important question for future studies.

Many groups have shown that immature and mature ILC2s cultured in media containing IL-7 and IL-2 combined with either IL-33 or IL-25 upregulate GATA3 and KLRG1 (respectively), secrete IL-5 and IL-13, and proliferate [15,20,21]. IL-33 can bind to ST2, leading to the induction of MYD88 and NFKB activation [22]. This signaling cascade is not only important for stimulating the secretion of IL-5 and IL-13 from ILC2s, but also is necessary for the migration of ILC2ps from the bone marrow to peripheral lymphoid organs [21]. Interestingly, thymic stromal lymphopoietin protein (TSLP)-deficient mice had a reduced frequency of ILC2p cells in the bone marrow compared to wild-type mice, whereas IL-33 and ST2-deficient mice had an increase in the frequency of ILC2p in the bone marrow compared to WT mice. ILC2p cells from IL33^−/−^ mice had enhanced CXCR4, a chemokine that retains leukocytes in the bone marrow, which was reversed following treatment with recombinant IL-33. These data suggest that TSLP is necessary for the development of ILC2p, but IL-33 is required for the migration of ILC2p, leaving the bone marrow in a CXCR4-dependent manner [21].

## 4. Regulation of ILC2

Regarding the cytokines that drive ILC2-associated mucosal inflammation, IL-33, IL-25, and TSLP can all activate ILC2s. The roles of these cytokines in ILC2 activation during helminth infection will be discussed in more detail later, in the context of epithelial cell-ILC2 crosstalk during helminth infection. However, it is important to note that IL-25 and IL-33 act in different ways and on distinct ILC2 subsets. While IL-25 can expand IL-13-producing “natural” ILC2s, it was also recently shown to expand a KLRG1^Hi^ population of “inflammatory” ILC2s that can produce IL-17 in the context of a *Candida albicans* infection. [23,24]. These cells respond to IL-25 but not IL-33 alone, exhibit plasticity between ILC2 and ILC3 functions, and can persist at the initial site of inflammation and produce type 2 cytokines long-term [25,26]. IL-33 has largely been shown to activate natural, type 2 cytokine-producing ILCs, but can also synergize leukotrienes and free fatty acids during type 2 inflammation in the airway to enhance ILC2 function [27,28]. That different signals or combinations of signals can so drastically alter ILC2 function suggests that ILC2s must be tightly controlled, and that negative regulatory mechanisms must also exist to prevent aberrant ILC2 function.

**Figure 1:**
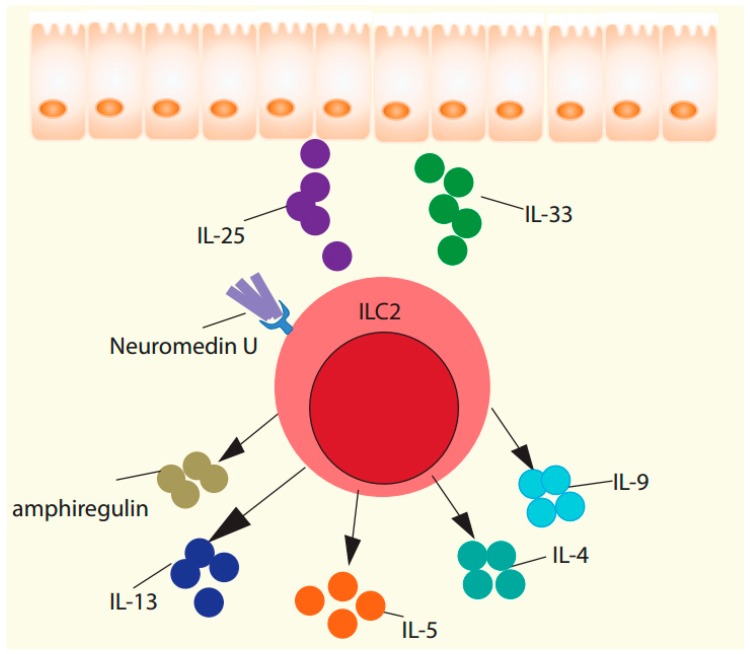
Biological importance of ILC2 in helminth immunity.

One such mechanism is through type I and II interferons (namely IFN-β and IFN-γ), which both suppressed proliferation and cytokine production by ILC2s [25,29]. Interestingly, while IFN-γ suppressed tissue-resident, natural ILC2 responses, it did not suppress inflammatory ILC2 responses, emphasizing the importance of signal integration and context in ILC2 regulation [25]. In addition to interferons, IL-27 also exhibits a suppressive effect on ILC2s [25,29]. In the absence of IL-27, ILC2s accumulate to a greater extent in response to inflammation in the lung. Ex vivo, the addition of IL-27 to ILC2s stimulated with IL-33 and IL-7 led to a decrease in IL-5 and IL-13 expression and secretion, suggesting that IL-27 directly represses ILC2 (Figure 1) [30]. Suppression of ILC2s by both interferons and IL-27 was dependent on STAT1 signaling [25,29]. These mechanisms may be important for curtailing ILC2 activation in contexts where unchecked functions would otherwise be antagonistic to ongoing immunity or even pathogenic to host tissue.

In addition to immunologic signals, ILC2s also sense metabolic signals, such as micronutrient availability, at tissue sites [31,32]. In the small intestine, ILC2s preferentially develop over ILC3s when Vitamin A and its derivative, retinoic acid, are diminished or absent [31]. This predominance of ILC2s in the small intestine confers enhanced immunity to helminth infection while exacerbating enteric bacterial infections [31]. Further work in this area shows that ILC2s thrive in this environment by increasing fatty acid acquisition and metabolism [32]. While there may be other metabolic signals that control ILC2 function, this mechanism of enhanced fatty acid oxidation in response to nutrient deprivation may have important implications for ILC2 function in human diseases and infections in regions of the world where malnutrition is prevalent.

Although ILC2s have drawn considerable interest within the past decade, there are many outstanding questions pertaining to tissue-specific gene expression patterns and biological roles of ILC2 depending on their location [21,33]. It is still unknown whether there are generalized suppressive mechanisms to limit ILC2 activation, such as regulatory ILCs (ILCregs) that produce IL-10, or conversion into different types by cytokines such as IL-27 and IL-12. These considerations are quite important due to the major role of ILC2 in the context of health and disease [12,34]. In particular, understanding how ILC2s are regulated in the context of a helminth infection will be necessary for improving existing anthelmintic drugs and developing new therapeutics to treat helminth infections.

## 5. ILC2 Expansion and Function at Sites of Helminth Infection

ILC2s were first discovered in gastrointestinal (GI) nematode infection models, and are now appreciated to be a predominant, early source of IL-13 following infection by these helminths, driving goblet cell hyperplasia, smooth muscle hypercontractility, mucus production, and, ultimately, worm clearance [3,4,6,35,36,37]. Infection with rodent hookworms *N. brasiliensis* or *Heligmosomoides polygyrus bakeri* induces the release of IL-25 and IL-33, which rapidly and robustly expands ILC2 populations in the small intestine [3,4,38,39]. In the lung during an *N. brasiliensis* infection, ILC2s are actively recruited in a CRTH2 (chemoattractant receptor homologous molecule expressed on Th2 cells)- and prostaglandin D2 (PGD2)-dependent manner [40]. Survival and cytokine production by ILC2 in the lung is also dependent on expression of Inducible T cell co-stimulator (ICOS) and ligation of this receptor by ICOS-L [41]. Furthermore, their expansion and IL-13 production in the lung is largely dependent on alarmin IL-33 and cytokine IL-9 during *Strongyloides venezuelensis* and *N. brasiliensis* infections [35,40,42,43,44,45].

While this work does not definitively show a necessary role for ILC2s in clearance, it suggests that these cells are important for IL-5, IL-13, and amphiregulin production and coordination of eosinophilia in the lung during infection [42,45]. The necessity of ILC2s during a GI nematode infection has been more clearly demonstrated using ILC2-deficient (*Rorα*^fl/sg^*Il7r*^Cre^) and ILC2 inducible deleter (ICOS-T) mice, in which clearance of the *N. brasiliensis* infection is significantly impaired [46]. While the necessity of ILC2s in other murine GI nematode models and human GI nematode infections remains to be demonstrated, work thus far indicates that ILC2 expansion and cytokine production are key events in the immune response to this family of helminths [47].

The role of ILC2s in infected tissues during schistosomiasis and filarial helminthiases is not as well studied as in GI nematode infections, but murine infection with filarial helminth *Litomosoides sigmodontis* or *Schistosoma mansoni* expands ILC2s locally [48]. In an *L. sigmodontis* infection, ILC2s increase at sites of adult worm infections within five days, and this population peaks before infections reach patency several weeks later. Furthermore, the high percentage of ILC2s that produce IL-5 during early infection suggests that these cells are an important source of this crucial cytokine and potentiate Th2 responses to *L. sigmodontis* that arise later during the infection [43,48]. Similarly, IL-13-producing ILCs are increased in adults with filarial infections (i.e., *Loa loa*, *Wuchereria bancrofti*, or *Onchocerca volvulus*), which, while correlative, suggests ILC2s may also be important cytokine-producers in human filariases (Table S1) [49].

In schistosomiasis, the role for ILC2s is more complicated. Expansion of ILC2s in the lungs and liver of *S. mansoni*-infected mice drives tissue fibrosis [24,44]. In the absence of either IL-25 signaling (*Il25*^−/−^ or *Il17rb*^−/−^ mice) or ILC2s (*Rorα*^sg/sg^ mice), pulmonary fibrosis during an *S. mansoni* infection was abrogated [24]. Similarly, hepatic fibrosis was abrogated in mice deficient in the IL-1R associated protein (IL-1R3), a subunit of the IL-33 receptor, and fibrosis was shown to be mediated in the liver by IL-33-responsive ILC2s [44]. In humans, while ILC2s do not increase in the peripheral blood adolescents and young adults infected with *Schistosoma haematobium* [50], humans with idiopathic pulmonary fibrosis exhibit increased percentages and numbers of ILC2s in bronchioalveolar lavage (BAL) fluid [24]. Thus, investigation of ILC2 populations at sites of fibrosis, such as the lung or liver, in human schistosomiasis patients may reveal a similar correlation between ILC2s and tissue damage in humans as has been demonstrated in mice. Surprisingly, however, investigations in children aged between 6 and 9 years old reveal that ILC2s are positively correlated with resolution of schistosomiasis. *S. haematobium*-infected children have significantly fewer circulating ILC2s than uninfected controls, and the administration of curative treatment (praziquantel) restores ILC2 numbers to those observed in children who tested negative for *S. haematobium* prior to treatment [50]. Though the exact function of ILC2s in human schistosomiasis remains to be investigated, data from both mice and humans suggests that ILC2s require tight regulation to maintain a balance between protection and host damage, and that their role in protection may change throughout human development.

One notable gap in our understanding of ILC2s at sites of helminth infection is their level of involvement in skin immune responses to penetrative helminth species, including hookworms like *N. brasiliensis* and systemic helminths like *S. mansoni*. Recent work by Obata-Ninomiya et al. demonstrates that basophil-derived IL-4 coordinates M2 macrophage-mediated trapping and killing of *N. brasiliensis* larvae in the skin upon secondary infection [51]. Unexpectedly, ILC2s were not shown to expand in the skin in response to an *N. brasiliensis* infection [51]. This finding differs from reports of ILC2 expansion at other sites of helminth infection, and is surprising given that IL-33, a key inducer of ILC2 expansion, is expressed both at baseline and during inflammation in the skin [52]. Whether skin ILC2s expand in response to other skin-penetrative worms, or whether their function is altered during skin penetration, is not clear. However, it is possible that the disparity in ILC2 expansion between the skin and other sites of infection (e.g., lung, GI tract) is due instead to functional difference between skin-resident ILC2s and those in other tissues. Single-cell transcriptomics of ILC2s reveals that these cells cluster distinctly based on their tissues of origin, and that skin ILC2s are uniquely programmed to respond to IL-18 as opposed to canonical type 2 signals like IL-25 and IL-33 (Ricardo-Gonzalez et al., 2018). This difference could explain why ILC2s were not observed to expand in the skin early following *N. brasiliensis* penetration. Alternatively, the lack of expansion of ILC2s could reflect suppression or evasion by the infective stage of the parasite. Though ILC2s have not been directly examined in this context, human Langerhans cells and dermal dendritic cells have been shown to remain quiescent and even downregulate innate immune genes when exposed to infective *Brugia malayi* larvae in vitro [53,54,55]. Investigating both the expansion and function of ILC2s in the context of different skin-penetrating helminth models will be necessary to understand whether ILC2s play a role in the immune response to larvae in the skin.

## 6. ILC2 Crosstalk with Epithelial Cells during Helminth Infection

Initial reports of ILC2s established that their expansion during helminth infection is driven largely by IL-17 family member IL-25 (IL-17E). However, IL-25 is expressed by intestinal epithelial cells, endothelial cells, type 2 CD4^+^ (Th2) cells, natural killer T cells (NKT), FcεR1-ligated mast cells, and eosinophils, and thus could come from a variety of cell types during helminth infection [3,4,38,39,56,57,58,59]. Interestingly, three independent groups recently demonstrated that intestinal epithelial cells, specifically rare, chemosensory Tuft cells, expand during worm infection and are necessary producers of IL-25 [38,39,60]. In the absence of Tuft cells, ILC2s fail to expand, leading to impaired clearance of both *N. brasiliensis* and *H. polygyrus*, but exogenous delivery of IL-25 is sufficient to induce worm expulsion [39]. Recently, it was demonstrated that Tuft cells themselves expand and produce IL-25 in response to microbial metabolite succinate, which is produced by both *N. brasiliensis* as well as protist *Trichomonas* [61,62]. Administration of succinate alone was sufficient to induce expansion of ILC2s in the small intestine in a succinate receptor (SUCNR1)-dependent manner [61,62]. While this receptor was not required for clearance of *N. brasiliensis*, expression of TRMP5, an ion channel important for taste signal transduction, was [61,62]. These results suggest that this Tuft cell-ILC2 circuit can be induced by the sensation of metabolites elicited at the site of the parasite infestation [61,62]. Moreover, once Tuft cells elicit ILC2 expansion, ILC2-derived IL-13 initiates a feed-forward loop that signals back to intestinal crypt cells via IL-4Ra to remodel the epithelium, promoting increased numbers of Tuft cells and goblet cells [39,60]. Since goblet cells produce mucus and resistins like Relm-β, that are necessary to expel worms from the intestine [63], this Tuft cell-ILC2 circuit is crucial for immunity against GI nematodes.

In addition to IL-25, IL-33 is also a key activator of ILC2s, especially in the lung but also in the GI tract [3,4,45]. Whole-body genetic deletion of IL-33 results in impaired clearance to *N. brasiliensis* in mice, which corresponds to reduced IL-13 production by ILC2s and, subsequently, reduced Relm-β production and eosinophilia [45]. However, the mechanism by which IL-33 is induced and released to act upon ILC2s during helminth infection remains unresolved. The current dogma is that IL-33 is released from epithelial cells upon worm-induced damage at barrier sites and cleaved into its bioactive form by cathepsins and other proteases, allowing it to bind its receptor on ILC2s and activate these cells [64], yet a requirement for epithelial-derived IL-33 for helminth clearance has not been demonstrated. This model is further complicated by recent reports indicating that a number of other cell types, including macrophages, mast cells, and dendritic cells, express IL-33 [64,65,66,67,68,69]. Recently, mast cells were shown to produce IL-33 during *H. polygyrus* infection, dependent on adenosine triphosphate ATP signaling through the P2X7 receptor [70]. Inhibition of P2X7 receptor signaling led to enhanced worm burdens, correlated with decreased expression of IL-33 in mast cells, while reconstitution of IL-33^−/−^ mice with IL-33-sufficient bone marrow reduced worm burdens and increased ILC2 frequencies [70]. That hematopoietic cells are important sources of IL-33 is further corroborated by work in the *Alternaria alternata* fungal allergen model, where IL-33 production by macrophages can lead to the activation of ILC2s in a group V phospholipase A2 (PLA2g5)-dependent manner [27,28]. Whether IL-33 production in other cell types is necessary for ILC2 activation and helminth clearance is not clear. Furthermore, it is not known whether the cells identified above must release IL-33 to activate ILC2s, or how IL-33 could be released from these cell types. Identifying critical source(s) of IL-33 and uncoupling the intra- and extra-cellular functions of IL-33 will be important for understanding how IL-33 regulates ILC2 activation and function during helminth infection.

In addition to producing IL-5, IL-13, and small amounts of IL-4, ILC2s are also key producers of IL-9 [35]. IL-9 was discovered as a cytokine produced by CD4^+^ T cells. IL-9 induces mastocytosis, eosinophila, mast cell infiltration, and mucus production indirectly via IL-4, IL-5, and IL-13 [71,72], and serves as a T cell and mast cell survival and growth factor [73,74]. Recently, ILC2s were found to produce IL-9 as well, and during an *N. brasiliensis* infection IL-9-producing ILC2s outnumber IL-9 producing CD4^+^ T cells 5–10-fold in the lung [35]. Furthermore, autocrine IL-9 signaling on ILC2s is important for their expansion and production of IL-5, IL-13, and epidermal growth factor receptor (EGFR)-ligand amphiregulin in the lung [35,36]. When IL-9R is genetically ablated, ILC2 but not CD4^+^ T cell expansion in the lung during *N. brasiliensis* infection is diminished, and IL-5, IL-13, and amphiregulin are significantly reduced [35]. Since ILC2-derived amphiregulin plays an important role in epithelial regeneration and tissue repair following helminth-induced tissue damage, loss of IL-9 signaling impairs lung tissue repair following an *N. brasiliensis* infection and ultimately delays worm clearance [35]. Furthermore, alveolar type II cell-derived IL-33 and TSLP are necessary to induce interferon regulatory factor 4(IRF4)-driven IL-9 production by ILC2s [36]. Thus, IL-9 is upregulated in ILC2s in response to epithelial damage and signals in an autocrine manner to induce IL-5, IL-13, and amphiregulin. These signals feedback on the epithelium to induce mucus production, smooth muscle hypercontraction, and barrier restitution during helminth infection.

**Figure 2:**
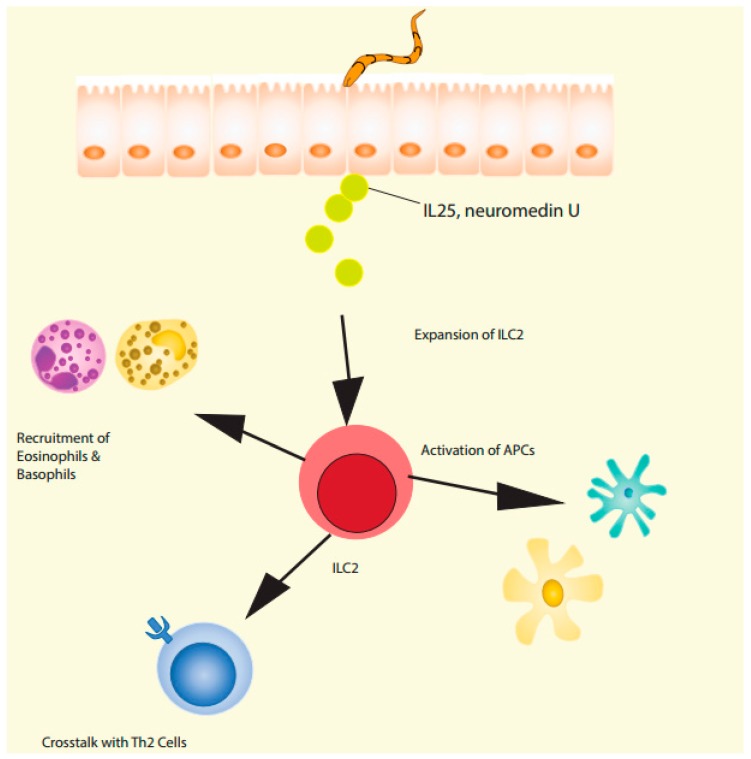
Cytokine circuits of ILC2 in helminth infection.

ILC2 crosstalk with CD4^+^ T cells during helminth infection, in addition to their crosstalk with epithelial cells. ILC2s also regulate and receive feedback from CD4^+^ T cells during helminth infections. CD4^+^ T cells are requisite for immunity against helminth infections in both mice and humans, but the mechanisms underlying their activation, function, and maintenance during these infections are not well understood [75,76,77,78]. However, emerging evidence suggests that ILC2–CD4^+^ T cell crosstalk in the context of a GI nematode infection is crucial for CD4^+^ T cell activation and function, as well as for worm clearance. For example, when ILC2s are genetically ablated, IL-5 and IL-13 production by CD4^+^ T cells in the MLN drops dramatically, and mice fail to expel *N. brasiliensis* [46]. In the lung, CD4^+^ T cells and ILC2s cooperate to promote M2 macrophage activation and M2-mediated larval trapping and killing. While ILC2 production of IL-13 alone appears to be sufficient to support this macrophage population, ILC2s depend on IL-2 from CD4^+^ T cells to survive and mediate these effects during infection [46,79]. Conversely, T_H_2 differentiation relies on ILC2-derived IL-4 during an *H. polygyrus* infection [80]. Thus, ILC2s and CD4^+^ T cells are interdependent in responding to helminth infections (Figure 2).

Interestingly, ILC2s have recently been shown to express major histocompatibility complex class II (MHCII), which is required for antigen presentation to CD4^+^ T cells [46,81]. Indeed MHCII-bearing ILC2s can present antigen to and expand CD4^+^ T cells in vitro, and their expression of the co-stimulatory molecule OX40L specifically elicits the expansion of T_h_2s and Tregs in response to IL-33 during an *N. brasiliensis* infection [46,81]. MHCII-mediated interactions between CD4^+^ T cells and ILC2s also impact on ILC2 function, as IL-2 is upregulated in ILC2-activated antigen-specific CD4^+^ T cells and is necessary for ILC2s to upregulate type 2 cytokines in vitro [46]. Moreover, loss of either MHCII or OX40L in ILC2s leads to the impaired clearance of *N. brasiliensis*, highlighting the importance of this axis in type 2 immune responses to parasitic nematodes [46,81].

## 7. Conclusions

While there has been considerable interest in ILC2 ontogeny, cytokine production, and regulation in the years since their discovery, there remains a paucity of information regarding the intercellular communicative networks that restrain and elicit ILC2 function. Cytokine-mediated crosstalk between epithelial barriers and ILC2s is undoubtedly important, but how ILC2s provide feedback to various epithelial cell lineages such as Tuft cells remains an open area of exploration. Moreover, the chemosensory cues that direct ILC2s to localize to specific niches for survival, expansion and cytokine production all remain areas that are, to date, largely obscure. Additionally, while signals such as IL-25 and IL-33 are known to be important for expanding ILC2s at sites like the gut and the lung, the requisite cellular source(s) of IL-25 and IL-33 have not yet been identified, and the regulatory mechanisms controlling IL-33 release remain poorly understood. Emerging evidence indicates that ILC2s can also interface with sensory nerve fibers through multiple mechanisms, including neuromedin U and acetylcholine [82]. However, whether, and if so, how ILC2 expansion reciprocally influences sensory nerve fibers and nervous system biology is not well understood. Improving our understanding of events that occur both upstream and downstream of ILC2 activation will inform not only basic research but also the design of therapeutics targeting ILC2s during helminth infection.

In addition to their crosstalk with epithelial barriers, ILC2s also communicate with other cells of the immune system. During helminth infection, ILC2s orchestrate the recruitment of eosinophils and mast cells and, in collaboration with CD4^+^ T cells, activate M2 macrophage subset development. While some work has shown that ILC2–CD4^+^ T cell crosstalk occurs in an MHCII-dependent manner, the antigen repertoire that ILC2s present to CD4^+^ T cells is not well characterized. Understanding how, where, and when ILC2s take up antigen will be important for understanding how CD4^+^ T cells recognize helminths and how vaccines can elicit better CD4^+^ T cell responses through this axis. Certainly, there is much work to be done in years to come that could have an important impact on human health and disease.

## Supplementary Materials

Supplementary materials can be found at https://www.mdpi.com/1422-0067/20/9/2276/s1.

## References

[1] Nair M.G., Guild K.J., Artis D. (2006). Novel effector molecules in type 2 inflammation: Lessons drawn from helminth infection and allergy. J. Immunol..

[2] Hung L.Y., Oniskey T.K., Sen D., Krummel M.F., Vaughan A.E., Cohen N.A., Herbert D.R. (2018). Trefoil Factor 2 Promotes Type 2 Immunity and Lung Repair through Intrinsic Roles in Hematopoietic and Nonhematopoietic Cells. Am. J. Pathol..

[3] Fallon P.G., Ballantyne S.J., Mangan N.E., Barlow J.L., Dasvarma A., Hewett D.R., McIlgorm A., Jolin H.E., McKenzie A.N. (2006). Identification of an interleukin (IL)-25-dependent cell population that provides IL-4, IL-5, and IL-13 at the onset of helminth expulsion. J. Exp. Med..

[4] Neill D.R., Wong S.H., Bellosi A., Flynn R.J., Daly M., Langford T.K., Bucks C., Kane C.M., Fallon P.G., Pannell R. (2010). Nuocytes represent a new innate effector leukocyte that mediates type-2 immunity. Nature.

[5] Price A.E., Liang H.E., Sullivan B.M., Reinhardt R.L., Eisley C.J., Erle D.J., Locksley R.M. (2010). Systemically dispersed innate IL-13-expressing cells in type 2 immunity. Proc. Natl. Acad. Sci. USA.

[6] Moro K., Yamada T., Tanabe M., Takeuchi T., Ikawa T., Kawamoto H., Furusawa J., Ohtani M., Fujii H., Koyasu S. (2010). Innate production of T(H)2 cytokines by adipose tissue-associated c-Kit(+)Sca-1(+) lymphoid cells. Nature.

[7] Camelo A., Barlow J.L., Drynan L.F., Neill D.R., Ballantyne S.J., Wong S.H., Pannell R., Gao W., Wrigley K., Sprenkle J. (2012). Blocking IL-25 signalling protects against gut inflammation in a type-2 model of colitis by suppressing nuocyte and NKT derived IL-13. J. Gastroenterol..

[8] Ricardo-Gonzalez R.R., Van Dyken S.J., Schneider C., Lee J., Nussbaum J.C., Liang H.E., Vaka D., Eckalbar W.L., Molofsky A.B., Erle D.J. (2018). Tissue signals imprint ILC2 identity with anticipatory function. Nat. Immunol..

[9] Spits H., Artis D., Colonna M., Diefenbach A., Di Santo J.P., Eberl G., Koyasu S., Locksley R.M., McKenzie A.N., Mebius R.E. (2013). Innate lymphoid cells—A proposal for uniform nomenclature. Nat. Rev. Immunol..

[10] Robinette M.L., Fuchs A., Cortez V.S., Lee J.S., Wang Y., Durum S.K., Gilfillan S., Colonna M., Immunological Genome C. (2015). Transcriptional programs define molecular characteristics of innate lymphoid cell classes and subsets. Nat. Immunol..

[11] Lei A.H., Xiao Q., Liu G.Y., Shi K., Yang Q., Li X., Liu Y.F., Wang H.K., Cai W.P., Guan Y.J. (2018). ICAM-1 controls development and function of ILC2. J. Exp. Med..

[12] Li B.W.S., Beerens D., Brem M.D., Hendriks R.W. (2017). Characterization of Group 2 Innate Lymphoid Cells in Allergic Airway Inflammation Models in the Mouse. Methods Mol. Biol..

[13] Seillet C., Rankin L.C., Groom J.R., Mielke L.A., Tellier J., Chopin M., Huntington N.D., Belz G.T., Carotta S. (2014). Nfil3 is required for the development of all innate lymphoid cell subsets. J. Exp. Med..

[14] Klose C.S.N., Flach M., Mohle L., Rogell L., Hoyler T., Ebert K., Fabiunke C., Pfeifer D., Sexl V., Fonseca-Pereira D. (2014). Differentiation of type 1 ILCs from a common progenitor to all helper-like innate lymphoid cell lineages. Cell.

[15] Hoyler T., Klose C.S., Souabni A., Turqueti-Neves A., Pfeifer D., Rawlins E.L., Voehringer D., Busslinger M., Diefenbach A. (2012). The transcription factor GATA-3 controls cell fate and maintenance of type 2 innate lymphoid cells. Immunity.

[16] Scoville S.D., Freud A.G., Caligiuri M.A. (2018). Cellular pathways in the development of human and murine innate lymphoid cells. Curr. Opin. Immunol..

[17] Walker J.A., Oliphant C.J., Englezakis A., Yu Y., Clare S., Rodewald H.R., Belz G., Liu P., Fallon P.G., McKenzie A.N. (2015). Bcl11b is essential for group 2 innate lymphoid cell development. J. Exp. Med..

[18] Yagi R., Zhong C., Northrup D.L., Yu F., Bouladoux N., Spencer S., Hu G., Barron L., Sharma S., Nakayama T. (2014). The transcription factor GATA3 is critical for the development of all IL-7Ralpha-expressing innate lymphoid cells. Immunity.

[19] Wong S.H., Walker J.A., Jolin H.E., Drynan L.F., Hams E., Camelo A., Barlow J.L., Neill D.R., Panova V., Koch U. (2012). Transcription factor RORalpha is critical for nuocyte development. Nat. Immunol..

[20] Yang Q., Monticelli L.A., Saenz S.A., Chi A.W., Sonnenberg G.F., Tang J., De Obaldia M.E., Bailis W., Bryson J.L., Toscano K. (2013). T cell factor 1 is required for group 2 innate lymphoid cell generation. Immunity.

[21] Stier M.T., Zhang J., Goleniewska K., Cephus J.Y., Rusznak M., Wu L., Van Kaer L., Zhou B., Newcomb D.C., Peebles R.S. (2018). IL-33 promotes the egress of group 2 innate lymphoid cells from the bone marrow. J. Exp. Med..

[22] Molofsky A.B., Van Gool F., Liang H.E., Van Dyken S.J., Nussbaum J.C., Lee J., Bluestone J.A., Locksley R.M. (2015). Interleukin-33 and Interferon-gamma Counter-Regulate Group 2 Innate Lymphoid Cell Activation during Immune Perturbation. Immunity.

[23] Hams E., Armstrong M.E., Barlow J.L., Saunders S.P., Schwartz C., Cooke G., Fahy R.J., Crotty T.B., Hirani N., Flynn R.J. (2014). IL-25 and type 2 innate lymphoid cells induce pulmonary fibrosis. Proc. Natl. Acad. Sci. USA.

[24] Hams E., Locksley R.M., McKenzie A.N., Fallon P.G. (2013). Cutting edge: IL-25 elicits innate lymphoid type 2 and type II NKT cells that regulate obesity in mice. J. Immunol..

[25] Moro K., Kabata H., Tanabe M., Koga S., Takeno N., Mochizuki M., Fukunaga K., Asano K., Betsuyaku T., Koyasu S. (2016). Interferon and IL-27 antagonize the function of group 2 innate lymphoid cells and type 2 innate immune responses. Nat. Immunol..

[26] Huang Y., Guo L., Qiu J., Chen X., Hu-Li J., Siebenlist U., Williamson P.R., Urban J.F., Paul W.E. (2015). IL-25-responsive, lineage-negative KLRG1(hi) cells are multipotential ‘inflammatory’ type 2 innate lymphoid cells. Nat. Immunol..

[27] Yamaguchi M., Samuchiwal S.K., Quehenberger O., Boyce J.A., Balestrieri B. (2018). Macrophages regulate lung ILC2 activation via Pla2g5-dependent mechanisms. Mucosal Immunol..

[28] Von Moltke J., O’Leary C.E., Barrett N.A., Kanaoka Y., Austen K.F., Locksley R.M. (2017). Leukotrienes provide an NFAT-dependent signal that synergizes with IL-33 to activate ILC2s. J. Exp. Med..

[29] Duerr C.U., McCarthy C.D., Mindt B.C., Rubio M., Meli A.P., Pothlichet J., Eva M.M., Gauchat J.F., Qureshi S.T., Mazer B.D. (2016). Type I interferon restricts type 2 immunopathology through the regulation of group 2 innate lymphoid cells. Nat. Immunol..

[30] McHedlidze T., Kindermann M., Neves A.T., Voehringer D., Neurath M.F., Wirtz S. (2016). IL-27 suppresses type 2 immune responses in vivo via direct effects on group 2 innate lymphoid cells. Mucosal Immunol..

[31] Spencer S.P., Wilhelm C., Yang Q., Hall J.A., Bouladoux N., Boyd A., Nutman T.B., Urban J.F., Wang J., Ramalingam T.R. (2014). Adaptation of innate lymphoid cells to a micronutrient deficiency promotes type 2 barrier immunity. Science.

[32] Wilhelm C., Harrison O.J., Schmitt V., Pelletier M., Spencer S.P., Urban J.F., Ploch M., Ramalingam T.R., Siegel R.M., Belkaid Y. (2016). Critical role of fatty acid metabolism in ILC2-mediated barrier protection during malnutrition and helminth infection. J. Exp. Med..

[33] Gasteiger G., Fan X., Dikiy S., Lee S.Y., Rudensky A.Y. (2015). Tissue residency of innate lymphoid cells in lymphoid and nonlymphoid organs. Science.

[34] Li R., Jiang X.X., Zhang L.F., Liu X.M., Hu T.Z., Xia X.J., Li M., Xu C.X. (2017). Group 2 Innate Lymphoid Cells Are Involved in Skewed Type 2 Immunity of Gastric Diseases Induced by Helicobacter pylori Infection. Mediators Inflamm..

[35] Turner J.E., Morrison P.J., Wilhelm C., Wilson M., Ahlfors H., Renauld J.C., Panzer U., Helmby H., Stockinger B. (2013). IL-9-mediated survival of type 2 innate lymphoid cells promotes damage control in helminth-induced lung inflammation. J. Exp. Med..

[36] Mohapatra A., Van Dyken S.J., Schneider C., Nussbaum J.C., Liang H.E., Locksley R.M. (2016). Group 2 innate lymphoid cells utilize the IRF4-IL-9 module to coordinate epithelial cell maintenance of lung homeostasis. Mucosal Immunol..

[37] Zhao A., McDermott J., Urban J.F., Gause W., Madden K.B., Yeung K.A., Morris S.C., Finkelman F.D., Shea-Donohue T. (2003). Dependence of IL-4, IL-13, and nematode-induced alterations in murine small intestinal smooth muscle contractility on Stat6 and enteric nerves. J. Immunol..

[38] Howitt M.R., Lavoie S., Michaud M., Blum A.M., Tran S.V., Weinstock J.V., Gallini C.A., Redding K., Margolskee R.F., Osborne L.C. (2016). Tuft cells, taste-chemosensory cells, orchestrate parasite type 2 immunity in the gut. Science.

[39] Gerbe F., Sidot E., Smyth D.J., Ohmoto M., Matsumoto I., Dardalhon V., Cesses P., Garnier L., Pouzolles M., Brulin B. (2016). Intestinal epithelial tuft cells initiate type 2 mucosal immunity to helminth parasites. Nature.

[40] Wojno E.D., Monticelli L.A., Tran S.V., Alenghat T., Osborne L.C., Thome J.J., Willis C., Budelsky A., Farber D.L., Artis D. (2015). The prostaglandin D(2) receptor CRTH2 regulates accumulation of group 2 innate lymphoid cells in the inflamed lung. Mucosal Immunol..

[41] Maazi H., Patel N., Sankaranarayanan I., Suzuki Y., Rigas D., Soroosh P., Freeman G.J., Sharpe A.H., Akbari O. (2015). ICOS:ICOS-ligand interaction is required for type 2 innate lymphoid cell function, homeostasis, and induction of airway hyperreactivity. Immunity.

[42] Yasuda K., Muto T., Kawagoe T., Matsumoto M., Sasaki Y., Matsushita K., Taki Y., Futatsugi-Yumikura S., Tsutsui H., Ishii K.J. (2012). Contribution of IL-33-activated type II innate lymphoid cells to pulmonary eosinophilia in intestinal nematode-infected mice. Proc. Natl. Acad. Sci. USA.

[43] Ritter M., Tamadaho R.S., Feid J., Vogel W., Wiszniewsky K., Perner S., Hoerauf A., Layland L.E. (2017). IL-4/5 signalling plays an important role during Litomosoides sigmodontis infection, influencing both immune system regulation and tissue pathology in the thoracic cavity. Int. J. Parasitol..

[44] McHedlidze T., Waldner M., Zopf S., Walker J., Rankin A.L., Schuchmann M., Voehringer D., McKenzie A.N., Neurath M.F., Pflanz S. (2013). Interleukin-33-dependent innate lymphoid cells mediate hepatic fibrosis. Immunity.

[45] Hung L.Y., Lewkowich I.P., Dawson L.A., Downey J., Yang Y., Smith D.E., Herbert D.R. (2013). IL-33 drives biphasic IL-13 production for noncanonical Type 2 immunity against hookworms. Proc. Natl. Acad. Sci. USA.

[46] Oliphant C.J., Hwang Y.Y., Walker J.A., Salimi M., Wong S.H., Brewer J.M., Englezakis A., Barlow J.L., Hams E., Scanlon S.T. (2014). MHCII-mediated dialog between group 2 innate lymphoid cells and CD4(+) T cells potentiates type 2 immunity and promotes parasitic helminth expulsion. Immunity.

[47] Nausch N., Mutapi F. (2018). Group 2 ILCs: A way of enhancing immune protection against human helminths?. Parasite Immunol..

[48] Boyd A., Killoran K., Mitre E., Nutman T.B. (2015). Pleural cavity type 2 innate lymphoid cells precede Th2 expansion in murine Litomosoides sigmodontis infection. Exp. Parasitol..

[49] Boyd A., Ribeiro J.M., Nutman T.B. (2014). Human CD117 (cKit)+ innate lymphoid cells have a discrete transcriptional profile at homeostasis and are expanded during filarial infection. PLoS ONE.

[50] Nausch N., Appleby L.J., Sparks A.M., Midzi N., Mduluza T., Mutapi F. (2015). Group 2 innate lymphoid cell proportions are diminished in young helminth infected children and restored by curative anti-helminthic treatment. PLoS Negl. Trop. Dis..

[51] Obata-Ninomiya K., Ishiwata K., Tsutsui H., Nei Y., Yoshikawa S., Kawano Y., Minegishi Y., Ohta N., Watanabe N., Kanuka H. (2013). The skin is an important bulwark of acquired immunity against intestinal helminths. J. Exp. Med..

[52] Seltmann J., Roesner L.M., von Hesler F.W., Wittmann M., Werfel T. (2015). IL-33 impacts on the skin barrier by downregulating the expression of filaggrin. J. Allergy Clin. Immunol..

[53] Boyd A., Bennuru S., Wang Y., Sanprasert V., Law M., Chaussabel D., Nutman T.B., Semnani R.T. (2013). Quiescent innate response to infective filariae by human Langerhans cells suggests a strategy of immune evasion. Infect. Immun..

[54] Cotton R.N., McDonald-Fleming R., Boyd A., Spates K., Nutman T.B., Tolouei Semnani R. (2015). Brugia malayi infective larvae fail to activate Langerhans cells and dermal dendritic cells in human skin. Parasite Immunol..

[55] Semnani R.T., Venugopal P.G., Leifer C.A., Mostbock S., Sabzevari H., Nutman T.B. (2008). Inhibition of TLR3 and TLR4 function and expression in human dendritic cells by helminth parasites. Blood.

[56] Zaph C., Du Y., Saenz S.A., Nair M.G., Perrigoue J.G., Taylor B.C., Troy A.E., Kobuley D.E., Kastelein R.A., Cua D.J. (2008). Commensal-dependent expression of IL-25 regulates the IL-23-IL-17 axis in the intestine. J. Exp. Med..

[57] Iwakura Y., Ishigame H., Saijo S., Nakae S. (2011). Functional specialization of interleukin-17 family members. Immunity.

[58] Fort M.M., Cheung J., Yen D., Li J., Zurawski S.M., Lo S., Menon S., Clifford T., Hunte B., Lesley R. (2001). IL-25 induces IL-4, IL-5, and IL-13 and Th2-associated pathologies in vivo. Immunity.

[59] Ikeda K., Nakajima H., Suzuki K., Kagami S., Hirose K., Suto A., Saito Y., Iwamoto I. (2003). Mast cells produce interleukin-25 upon Fc epsilon RI-mediated activation. Blood.

[60] Von Moltke J., Ji M., Liang H.E., Locksley R.M. (2016). Tuft-cell-derived IL-25 regulates an intestinal ILC2-epithelial response circuit. Nature.

[61] Schneider C., O’Leary C.E., von Moltke J., Liang H.E., Ang Q.Y., Turnbaugh P.J., Radhakrishnan S., Pellizzon M., Ma A., Locksley R.M. (2018). A Metabolite-Triggered Tuft Cell-ILC2 Circuit Drives Small Intestinal Remodeling. Cell.

[62] Nadjsombati M.S., McGinty J.W., Lyons-Cohen M.R., Jaffe J.B., DiPeso L., Schneider C., Miller C.N., Pollack J.L., Nagana Gowda G.A., Fontana M.F. (2018). Detection of Succinate by Intestinal Tuft Cells Triggers a Type 2 Innate Immune Circuit. Immunity.

[63] Herbert D.R., Yang J.Q., Hogan S.P., Groschwitz K., Khodoun M., Munitz A., Orekov T., Perkins C., Wang Q., Brombacher F. (2009). Intestinal epithelial cell secretion of RELM-beta protects against gastrointestinal worm infection. J. Exp. Med..

[64] Wills-Karp M., Rani R., Dienger K., Lewkowich I., Fox J.G., Perkins C., Lewis L., Finkelman F.D., Smith D.E., Bryce P.J. (2012). Trefoil factor 2 rapidly induces interleukin 33 to promote type 2 immunity during allergic asthma and hookworm infection. J. Exp. Med..

[65] Furukawa S., Moriyama M., Miyake K., Nakashima H., Tanaka A., Maehara T., Iizuka-Koga M., Tsuboi H., Hayashida J.N., Ishiguro N. (2017). Interleukin-33 produced by M2 macrophages and other immune cells contributes to Th2 immune reaction of IgG4-related disease. Sci. Rep..

[66] Watanabe T., Yamashita K., Arai Y., Minaga K., Kamata K., Nagai T., Komeda Y., Takenaka M., Hagiwara S., Ida H. (2017). Chronic Fibro-Inflammatory Responses in Autoimmune Pancreatitis Depend on IFN-alpha and IL-33 Produced by Plasmacytoid Dendritic Cells. J. Immunol..

[67] Qi F., Wang D., Liu J., Zeng S., Xu L., Hu H., Liu B. (2015). Respiratory macrophages and dendritic cells mediate respiratory syncytial virus-induced IL-33 production in TLR3- or TLR7-dependent manner. Int. Immunopharmacol..

[68] Hsu C.L., Bryce P.J. (2012). Inducible IL-33 expression by mast cells is regulated by a calcium-dependent pathway. J. Immunol..

[69] Tjota M.Y., Hrusch C.L., Blaine K.M., Williams J.W., Barrett N.A., Sperling A.I. (2014). Signaling through FcRgamma-associated receptors on dendritic cells drives IL-33-dependent TH2-type responses. J. Allergy Clin. Immunol..

[70] Shimokawa C., Kanaya T., Hachisuka M., Ishiwata K., Hisaeda H., Kurashima Y., Kiyono H., Yoshimoto T., Kaisho T., Ohno H. (2017). Mast Cells Are Crucial for Induction of Group 2 Innate Lymphoid Cells and Clearance of Helminth Infections. Immunity.

[71] Schmitt E., Van Brandwijk R., Van Snick J., Siebold B., Rude E. (1989). TCGF III/P40 is produced by naive murine CD4+ T cells but is not a general T cell growth factor. Eur. J. Immunol..

[72] Temann U.A., Ray P., Flavell R.A. (2002). Pulmonary overexpression of IL-9 induces Th2 cytokine expression, leading to immune pathology. J. Clin. Investig..

[73] Hultner L., Moeller J., Schmitt E., Jager G., Reisbach G., Ring J., Dormer P. (1989). Thiol-sensitive mast cell lines derived from mouse bone marrow respond to a mast cell growth-enhancing activity different from both IL-3 and IL-4. J. Immunol..

[74] Hultner L., Druez C., Moeller J., Uyttenhove C., Schmitt E., Rude E., Dormer P., Van Snick J. (1990). Mast cell growth-enhancing activity (MEA) is structurally related and functionally identical to the novel mouse T cell growth factor P40/TCGFIII (interleukin 9). Eur. J. Immunol..

[75] Urban J.F., Katona I.M., Finkelman F.D. (1991). Heligmosomoides polygyrus: CD4+ but not CD8+ T cells regulate the IgE response and protective immunity in mice. Exp. Parasitol..

[76] Fowell D.J., Magram J., Turck C.W., Killeen N., Locksley R.M. (1997). Impaired Th2 subset development in the absence of CD4. Immunity.

[77] Hotez P.J., Brindley P.J., Bethony J.M., King C.H., Pearce E.J., Jacobson J. (2008). Helminth infections: The great neglected tropical diseases. J. Clin. Investig..

[78] Bourke C.D., Maizels R.M., Mutapi F. (2011). Acquired immune heterogeneity and its sources in human helminth infection. Parasitology.

[79] Bouchery T., Kyle R., Camberis M., Shepherd A., Filbey K., Smith A., Harvie M., Painter G., Johnston K., Ferguson P. (2015). ILC2s and T cells cooperate to ensure maintenance of M2 macrophages for lung immunity against hookworms. Nat. Commun..

[80] Pelly V.S., Kannan Y., Coomes S.M., Entwistle L.J., Ruckerl D., Seddon B., MacDonald A.S., McKenzie A., Wilson M.S. (2016). IL-4-producing ILC2s are required for the differentiation of TH2 cells following Heligmosomoides polygyrus infection. Mucosal Immunol..

[81] Halim T.Y.F., Rana B.M.J., Walker J.A., Kerscher B., Knolle M.D., Jolin H.E., Serrao E.M., Haim-Vilmovsky L., Teichmann S.A., Rodewald H.R. (2018). Tissue-Restricted Adaptive Type 2 Immunity Is Orchestrated by Expression of the Costimulatory Molecule OX40L on Group 2 Innate Lymphoid Cells. Immunity.

[82] Quatrini L., Vivier E., Ugolini S. (2018). Neuroendocrine regulation of innate lymphoid cells. Immunol. Rev..

